# Tophaceous gout-related carpal tunnel syndrome and flexor tendon involvement: a systematic review

**DOI:** 10.3389/fsurg.2026.1948883

**Published:** 2026-10-02

**Authors:** Ali Can Gunenc, Mehmet Sefik Oruc

**Affiliations:** Department of Plastic, Reconstructive and Aesthetic Surgery, Faculty of Medicine, İstanbul Aydın University, İstanbul, Türkiye

**Keywords:** carpal tunnel syndrome, flexor tendon, gout, median nerve compression, systematic review, tenosynovitis, tophaceous gout

## Abstract

**Background:**

Tophaceous gout is an uncommon but clinically important cause of secondary carpal tunnel syndrome (CTS), with lesions ranging from diffuse flexor tenosynovitis to intratendinous destruction and direct median nerve involvement.

**Methods:**

PubMed/MEDLINE, Scopus, and Web of Science Core Collection were searched through 23 July 2026, supplemented by Google Scholar, publisher-page verification, and citation searching. Human primary reports explicitly attributing CTS or wrist-level median neuropathy to gout, tophus, or monosodium urate deposition were eligible. Two reviewers independently screened records. Fifty-seven reports (27 complete/detailed and 30 limited clinical sources) formed the primary synthesis and underwent design-appropriate Joanna Briggs Institute appraisal; 12 bibliographic or translation-limited reports were retained as historical ancillary evidence. Primary reports were additionally classified *post hoc* by causal certainty.

**Results:**

Of 478 database records, 302 unique records were screened, and 69 canonical reports were included. The 57-report primary synthesis comprised 44 case reports or clinical-image reports, 12 case series, and one nonrandomized comparative study, representing at least 120 patients, 152 CTS wrists, and 146 gout-attributable wrists. Sex was reportable for 117 patients, of whom 111 (94.9%) were male. Causal certainty was Tier 1 (direct local confirmation) in 41 reports, Tier 2 (anatomically supported) in 5, and Tier 3 (explicit report-level attribution) in 11. *Post hoc* sensitivity analyses retaining Tier 1 + 2 (*n* = 46) and then Tier 1 only (*n* = 41) did not change the principal qualitative interpretations. Open decompression with lesion treatment was the most frequently reported operative pattern, but evidence remained insufficient to determine a preferred tendon procedure. JBI findings are reported by design; 481 item-level judgments are retained only as an audit total.

**Conclusion:**

Gout should be considered in atypical, acute, severe, recurrent, bilateral, or mass-associated CTS. In surgically treated reports, decompression was commonly combined with individualized management of tophaceous and tendon disease. The evidence is predominantly case-based and hypothesis-generating and is insufficient to define a validated treatment pathway or preferred tendon procedure.

## 1 Introduction

Carpal tunnel syndrome (CTS) is the most common entrapment neuropathy of the upper extremity. Although most cases are idiopathic, a secondary space-occupying or inflammatory cause should be considered when symptoms are acute, rapidly progressive, severe, recurrent after release, bilateral, associated with a palpable mass, or accompanied by restricted tendon excursion (1).

Monosodium urate deposition can increase the contents of the carpal tunnel through synovial hypertrophy, diffuse tenosynovitis, discrete tophaceous masses, intratendinous enlargement, or involvement of the tunnel floor and carpal structures. Rare reports describe direct deposition within the subepineurial plane of the median nerve. The published literature spans 1958–2026 and is dominated by case reports and small observational series (2–70).

This clinical problem is relevant to reconstructive and hand surgery because adequate treatment may require more than division of the transverse carpal ligament. Surgeons may need to inspect the median nerve, excise or debulk tophaceous material, perform tenosynovectomy or adhesiolysis, assess tendon integrity, and choose among tendon-preserving trimming, selective excision, and reconstruction. Because surgery does not treat the underlying metabolic disease, sustained urate control remains essential (71).

Previous syntheses have generally emphasized isolated contemporary cases and have not fully integrated historical reports, recent imaging observations, tendon-focused series, and structured methodological appraisal. This review therefore aimed to characterize: (1) clinical presentation and diagnostic red flags; (2) anatomical distribution of gout within the carpal tunnel and flexor apparatus; (3) the contribution of electrodiagnosis and imaging; (4) operative and nonoperative management; (5) outcomes, recurrence, and complications; and (6) the completeness and methodological limitations of the available evidence.

## 2 Methods

### 2.1 Study design, review question, and reporting framework

This systematic review was reported according to PRISMA 2020 (72). The review question was: among human patients with CTS or wrist-level median nerve compression (population), what clinical, anatomical, diagnostic, management, and outcome patterns have been reported when gout, tophus, or monosodium urate deposition is the causal condition (exposure) across primary clinical study designs? A comparator was not required because the literature is predominantly descriptive.

### 2.2 Systematic review protocol and registration

The review was not prospectively registered. Before the final synthesis, the operational methods were specified and date-stamped in an internal audit workbook, including the review question, eligibility criteria, information sources, deduplication and report-linkage rules, independent screening, second-reviewer verification of all extracted and not-reported fields, source-availability tiers, design-appropriate Joanna Briggs Institute appraisal tools, and the planned descriptive synthesis. Clarifications arising during duplicate linkage or source verification were documented with report-specific rationales. During peer review, a *post hoc* causal-certainty classification and qualitative sensitivity analysis were added to distinguish the strength of causal confirmation; these additions did not alter report eligibility, synthesis role, or the extracted clinical data. In response to a subsequent peer-review request, a conservative quantitative reporting-frequency synthesis and a focused assessment of potential cohort overlap were also added; these additions likewise did not alter report eligibility, synthesis role, or the extracted clinical data. The submission files provide the final search, selection, extraction, appraisal, and sensitivity-analysis outputs. No unpublished patient material, institutional case, submitted manuscript, or personal communication was included.

### 2.3 Participants, exposures/interventions, comparators, outcomes, and study designs (PICOS)

Population: human patients with CTS or wrist-level median nerve compression. Exposure/intervention: gout, tophus, or monosodium urate deposition explicitly identified as the causal condition; reported management interventions included medical urate control, carpal tunnel decompression, tophus excision or debulking, tenosynovectomy, tendon-preserving trimming, selective tendon excision, reconstruction, and rehabilitation. Comparator: no comparator was required because most evidence was descriptive; within-patient or between-group comparisons were retained when reported. Outcomes: presentation, electrodiagnostic and imaging findings, anatomical distribution, operative or pathological findings, treatment, functional outcomes, follow-up, recurrence, and adverse events. Study designs: primary clinical case reports, clinical image reports, case series, retrospective imaging or operative series, and nonrandomized comparative series.

### 2.4 Data sources and search strategy

PubMed/MEDLINE, Scopus, and Web of Science Core Collection were searched from database inception through 23 July 2026 without date or language restrictions. The prespecified search plan did not include Embase, which was not available through the authors’ institutional subscriptions during the review period. The strategy combined terms for CTS or median nerve compression with terms for gout, tophus, tophaceous disease, or monosodium urate, together with wrist, hand, flexor tendon, or tenosynovitis terms. Database-specific strategies and record counts are provided in Supplementary Table S1.

Supplementary searching included structured Google Scholar searches, publisher page verification, and backward and forward citation searching of eligible reports and relevant reviews. Dynamic Google Scholar result counts were not included in the PRISMA denominator; only unique peer-reviewed candidate reports entered into the screening log were counted.

### 2.5 Eligibility criteria

Eligible evidence consisted of human primary clinical reports meeting two minimum causal-eligibility requirements: (1) definite CTS or wrist-level median neuropathy as diagnosed in the primary report and (2) explicit attribution of that neuropathy to gout, tophus, or monosodium urate deposition at the carpal tunnel or wrist. A history of gout, hyperuricemia, or gout elsewhere in the hand or wrist without an explicit causal link to the median neuropathy was insufficient. Eligible designs included case reports, clinical image reports, case series, retrospective imaging or operative series, and nonrandomized comparative series. Reports could provide data on clinical presentation, electrodiagnostic findings, imaging, anatomical localization, operative or pathological findings, medical treatment, rehabilitation, follow-up, recurrence, or adverse events.

To distinguish eligibility from the strength of causal confirmation, the 57 primary-synthesis reports were classified after extraction using a *post hoc* three-tier operational framework. Tier 1 (direct local confirmation) required direct operative visualization or removal of the local gouty compressive lesion and/or histopathological or crystal confirmation at the wrist/carpal tunnel. Tier 2 (anatomically supported) required objective CTS or median neuropathy plus imaging-localized gout or tophi anatomically linked to median-nerve compression without direct local pathological or crystal confirmation in the accessible source. Tier 3 (explicit report-level attribution) met the minimum causal-eligibility criterion but the accessible source did not expose direct local confirmation or sufficiently specific anatomical imaging confirmation. The tier described causal certainty rather than overall study quality and did not determine inclusion.

Reports were excluded if they contained no primary clinical data; did not establish definite CTS or wrist-level median nerve compression; described gout in the hand or wrist without a causal relationship to CTS; reported postoperative gout without evidence that gout caused the preoperative neuropathy; involved non-urate crystal deposition; lacked an extractable gout-related CTS subgroup; or represented a duplicate publication of the same clinical report. Preprints and conference abstracts without sufficient peer-reviewed clinical information were not included. MEDLINE indexing status, journal impact metrics, and journal prestige were not eligibility criteria; indexing status was not used as a proxy for study quality. These source-status rules are summarized in Supplementary Table S1F.

### 2.6 Study selection and report linkage

After database merging, records were deduplicated by DOI, PMID, title, authorship, and publication year. A second-pass linkage audit identified residual duplicates and alternate-language or alternate-title representations of the same report. The two reviewers (ACG and MSO) independently screened the 302 unique database records; before consensus, exact agreement was 79.8%, and Cohen's kappa was 0.626. ACG and MSO also independently screened the six unique supplementary candidates. Disagreements were resolved through discussion and consensus.

ACG and MSO independently assessed report-level eligibility using the best available source: a complete article, detailed or structured abstract, official publisher excerpt, indexed clinical text, or bibliographic record. Reports were retained only when the accessible source explicitly established a causal relationship between gout or tophi and CTS or wrist-level median nerve compression. Two duplicate report representations were consolidated so that each clinical report appeared once in the canonical dataset. All final inclusion, exclusion, and linkage decisions were recorded with report-specific rationales.

Potential cohort overlap was assessed separately from duplicate-publication linkage where source detail permitted, using institution, authorship, recruitment period, patient and wrist counts, demographic characteristics, and intervention details. When clinically meaningful overlap could not be excluded, reports were not assumed to represent fully independent patient populations and aggregate counts were interpreted conservatively. These rules are summarized in Supplementary Table S1F.

### 2.7 Source-availability tiers and handling of missing information

To prevent limitations in source access from being mistaken for clinical evidence or methodological weakness, included reports were classified as: (1) complete or detailed source; (2) limited clinical source; or (3) bibliographic or translation-limited source. A limited clinical source was one for which no complete text was reviewed but an official abstract, publisher excerpt, or indexed clinical text was sufficient to establish eligibility and selected variables. The final source-access audit classified 27 reports as complete/detailed and 30 as limited clinical; these 57 reports formed the primary clinical synthesis and design-appropriate appraisal set. The 12 bibliographic or translation-limited reports were retained separately as historical ancillary evidence for verified report-level facts and conservative minimum counts and were not formally JBI-appraised. Unavailable variables were coded as not reported, excluded from domain-specific denominators, and not inferred from titles or indexing terms.

Because some historical series did not provide complete patient or CTS-subgroup denominators, aggregate counts are reported as conservative minimum estimates. Reports, patients, wrists, and definite gout-attributable wrists were maintained as separate units of analysis. Primary-synthesis estimates and full historical evidence-map estimates are reported separately.

### 2.8 Data extraction

A standardized extraction form captured citation, country, design, patient and wrist counts, sex, age, gout history, diabetes, laterality, acute or chronic course, CTS presentation, objective confirmation, imaging, anatomical gout site, specific flexor tendon involvement, diffuse tenosynovitis, direct median nerve involvement, treatment, tendon procedure, urate-lowering therapy, rehabilitation, follow-up, outcome, recurrence, adverse events, and key limitations. One reviewer entered each field, and the second reviewer independently verified every populated field and every field coded as not reported against the best available source. Discrepancies were resolved by consensus. Data were recorded exactly as reported; missing or inaccessible variables were not imputed.

The extraction dataset was canonicalized so that repeat extractions and duplicate report representations did not inflate counts. Reports, patients, CTS wrists, and explicitly gout-attributable wrists were maintained as separate units. Manuscript denominators, subgroup statements, tables, figures, and Supplementary Files were cross-checked against the locked canonical dataset before drafting.

### 2.9 Assessment of risk of bias and reporting quality

ACG and MSO appraised the 57 clinically appraisable reports with design-appropriate Joanna Briggs Institute (JBI) tools: the case-report checklist for 44 case reports or clinical-image reports, the case-series checklist for 12 case series, and the revised quasi-experimental checklist for the single nonrandomized comparative study (73–75). The comparative study was not pooled with the case-series appraisal because it explicitly compared tendon trimming with tendon resection.

Each item was rated Yes, No, Unclear, or Not applicable. Disagreements were resolved through discussion and consensus, and the final rationale was documented at item level. Source accessibility and JBI appraisal were treated as separate dimensions. For limited clinical sources, an item that could not be judged from the accessible abstract, publisher excerpt, or indexed clinical text was rated Unclear rather than No; therefore, Unclear denotes insufficient accessible reporting and should not be interpreted as confirmed methodological weakness or proof that the feature was absent. A No judgment was reserved for information supporting that the JBI criterion was not met. No numerical quality score, percentage threshold, or appraisal-based exclusion rule was applied. The 481 item-level judgments are retained as an audit total only and are not interpreted as a pooled cross-design quality score; design-specific matrices and summaries are the primary appraisal outputs.

### 2.10 Data analysis and synthesis

Meta-analysis was not appropriate because the evidence comprised heterogeneous case reports and small series with nonuniform anatomy, interventions, outcomes, and follow-up. Findings were synthesized narratively and descriptively. The 57 complete/detailed or limited clinical reports formed the primary clinical synthesis only to the extent supported by the accessible source. The 12 bibliographic or translation-limited reports formed a separate historical ancillary set and contributed verified report-level or conservative minimum-count information, but no unverified clinical, treatment, outcome, recurrence, complication, or JBI claims. *Post hoc* qualitative causal-certainty sensitivity analyses repeated the narrative synthesis after excluding Tier 3 reports (Tier 1 + 2, *n* = 46) and after restricting the synthesis to Tier 1 reports with direct local confirmation (*n* = 41). These analyses tested robustness of the narrative interpretation; no pooled effect or comparative sensitivity estimate was calculated. Figure 1 is a hypothesis-generating, nonvalidated evidence map and is not presented as a treatment algorithm, clinical pathway, or set of treatment indications.

**Figure 1:**
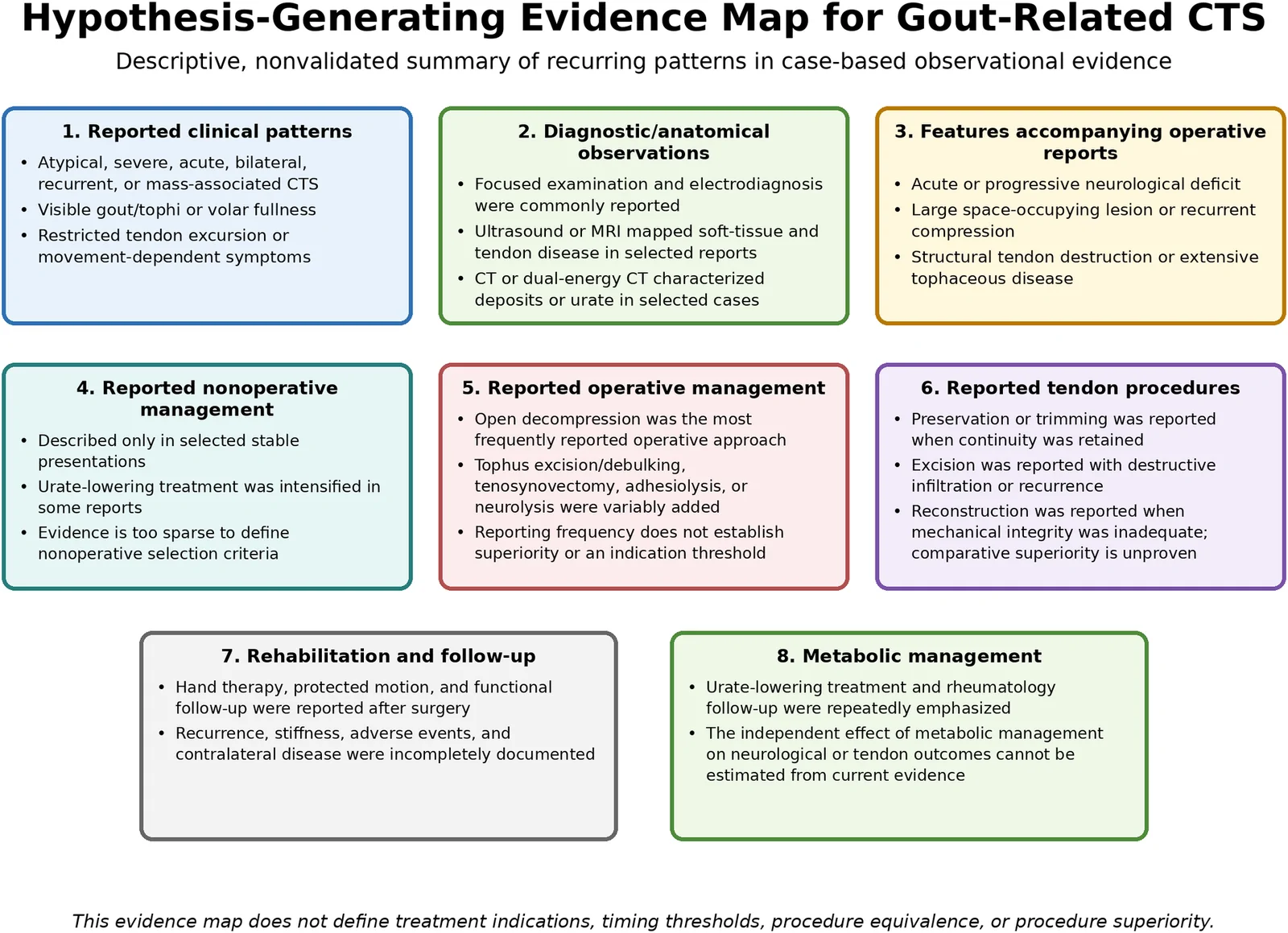
Hypothesis-generating evidence map for gout-related carpal tunnel syndrome. This nonvalidated map summarizes recurring observations in the case-based literature across clinical presentation, diagnostic/anatomical assessment, operative and nonoperative management, tendon procedures, rehabilitation, and metabolic care. It does not define treatment indications, timing thresholds, procedure equivalence, or procedure superiority..

To improve the transparency of the descriptive synthesis, conservative quantitative reporting frequencies were additionally calculated across the 57-report primary clinical synthesis. Missing or inaccessible fields coded as not reported (NR) were not imputed. Denominators were variable-specific and defined at the patient or report level as appropriate; the unit of analysis is stated explicitly. Imaging modalities and tendon-procedure categories were considered non-mutually exclusive when more than one modality or procedure was reported. These frequencies were interpreted as minimum reporting counts rather than estimates of patient-level prevalence, treatment efficacy, recurrence incidence, or complication incidence. The quantitative synthesis is presented in Table 1, Panel B and Supplementary Table S3C.

**Table 1:** Evidence profile and quantitative descriptive synthesis of the included reports.

Panel A. Evidence profile	
Characteristic	Final evidence set
Publication years	1958–2026
Unique canonical reports	69
Case reports/clinical-image reports	55
Case series	13
Nonrandomized comparative studies	1
Primary clinical synthesis/appraisal set	57 reports: 44 case reports/clinical-image reports; 12 case series; 1 nonrandomized comparative study
Primary-set minimum patients	120
Primary-set CTS wrists	152
Primary-set wrists with explicit gout attribution	146
Historical ancillary reports	12; retained for verified historical facts and conservative minimum counts; not formally JBI-appraised
Full evidence-map minimum patients	133
Full evidence-map CTS wrists	165
Full evidence-map wrists with explicit gout attribution	159
Source availability	27 complete/detailed; 30 limited clinical; 12 bibliographic or translation-limited
Critical-appraisal output	Design-specific JBI matrices for 57 reports; 481 item judgments retained as an audit total only
Final appraisal judgments (audit total)	Yes 324; No 6; Unclear 141; Not applicable 10
Causal certainty within primary synthesis	Tier 1 direct local confirmation 41; Tier 2 anatomically supported 5; Tier 3 explicit report-level attribution 11
Causal-certainty sensitivity sets	Tier 1 + 2: 46 reports; Tier 1 only: 41 reports; qualitative robustness checks only

Panel B. Quantitative descriptive synthesis of the 57-report primary clinical set		
Domain	Variable	Explicit reporting/denominator
Demographics	Male sex among patients with reportable sex	111/117 patients (94.9%)
	Female sex among patients with reportable sex	6/117 patients (5.1%)
	Numeric age information	45/57 reports (78.9%); age range 20–88 years
Gout history/metabolic status	Gout/hyperuricemia duration or onset timing	18/57 reports (31.6%)
	Serum-urate status and/or patient-specific ULT/adherence information	34/57 reports (59.6%)
Clinical presentation	Bilateral CTS explicitly reported	20/57 reports (35.1%)
	Visible/peripheral tophi explicitly reported	9/57 reports (15.8%)
Diagnosis	Electrodiagnostic testing explicitly documented	19/57 reports (33.3%)
Imaging	At least one named imaging modality	31/57 reports (54.4%)
Imaging	MRI	15/57 reports (26.3%)
Imaging	Radiography	14/57 reports (24.6%)
Imaging	Ultrasonography	7/57 reports (12.3%)
Imaging	CT	5/57 reports (8.8%)
Imaging	Dual-energy or dual-source CT	2/57 reports (3.5%)
Confirmation	Local histological/microscopic-crystal/pathological confirmation	19/57 reports (33.3%)
Surgery	Carpal-tunnel release/decompression explicitly performed	34/57 reports (59.6%)
	Open/extended-open or mini-incision approach specified	18/34 decompression reports (52.9%)
	Decompression approach insufficiently specified	16/34 decompression reports (47.1%)
Tendon procedure	Any tendon- or tendon-sheath-directed procedure	22/57 reports (38.6%)
	Tenosynovectomy/synovectomy	12/22 tendon-procedure reports (54.5%)
	Debulking/trimming/debridement	10/22 tendon-procedure reports (45.5%)
	Tendon excision/resection	6/22 tendon-procedure reports (27.3%)
	Reconstruction/repair/tubularization	7/22 tendon-procedure reports (31.8%)
Follow-up	Quantitative follow-up duration	25/57 reports (43.9%); range 1–82 months
Recurrence	Reports with quantitative follow-up and explicit recurrence outcome	15/57 reports (26.3%)
	Postoperative CTS/tophaceous recurrence	2/15 evaluable reports (13.3% reporting proportion)
Adverse events	Explicitly assessable adverse-event/complication status	25/57 reports (43.9%)
	Post-treatment event/complication reported	7/25 assessable reports (28.0% reporting proportion)

Counts are conservative minimum reporting frequencies derived from the locked canonical extraction dataset. NR fields were not imputed and absence of reporting was not interpreted as absence of a feature. Unless otherwise stated, report-level rows use the 57-report primary clinical set as the denominator. Imaging and tendon-procedure subcategories are non-mutually exclusive. Recurrence and adverse-event proportions are report-level reporting proportions, not pooled patient- or wrist-level incidence estimates. A matching audit summary is provided in Supplementary Table S3C.

### 2.11 Use of generative artificial intelligence

The authors declare that generative artificial intelligence was used in the preparation of this manuscript. OpenAI ChatGPT (GPT-5.6 Thinking and GPT-5.6 Sol; OpenAI, San Francisco, CA, United States; accessed July–August 2026) was used for language editing, document organization, structured consistency checks, and assistance with the generation and visual refinement of schematic table and figure layouts. It was not used to make autonomous screening, eligibility, extraction, critical-appraisal, or interpretive decisions. Both authors verified all source linkages, data, judgments, interpretations, figures, and references and take full responsibility for the final content.

## 3 Results

### 3.1 Study selection

The database searches identified 478 records: 157 from PubMed/MEDLINE, 271 from Scopus, and 50 from Web of Science Core Collection. After removal of 176 duplicates, 302 unique database records were screened, and 217 were excluded. Eighty-five database reports and six unique supplementary peer-reviewed reports entered report-level eligibility assessment (*n* = 91). Twenty reports were excluded, and two duplicate report representations were consolidated, yielding 69 unique included reports (Figure 2; Supplementary Table S1).

**Figure 2:**
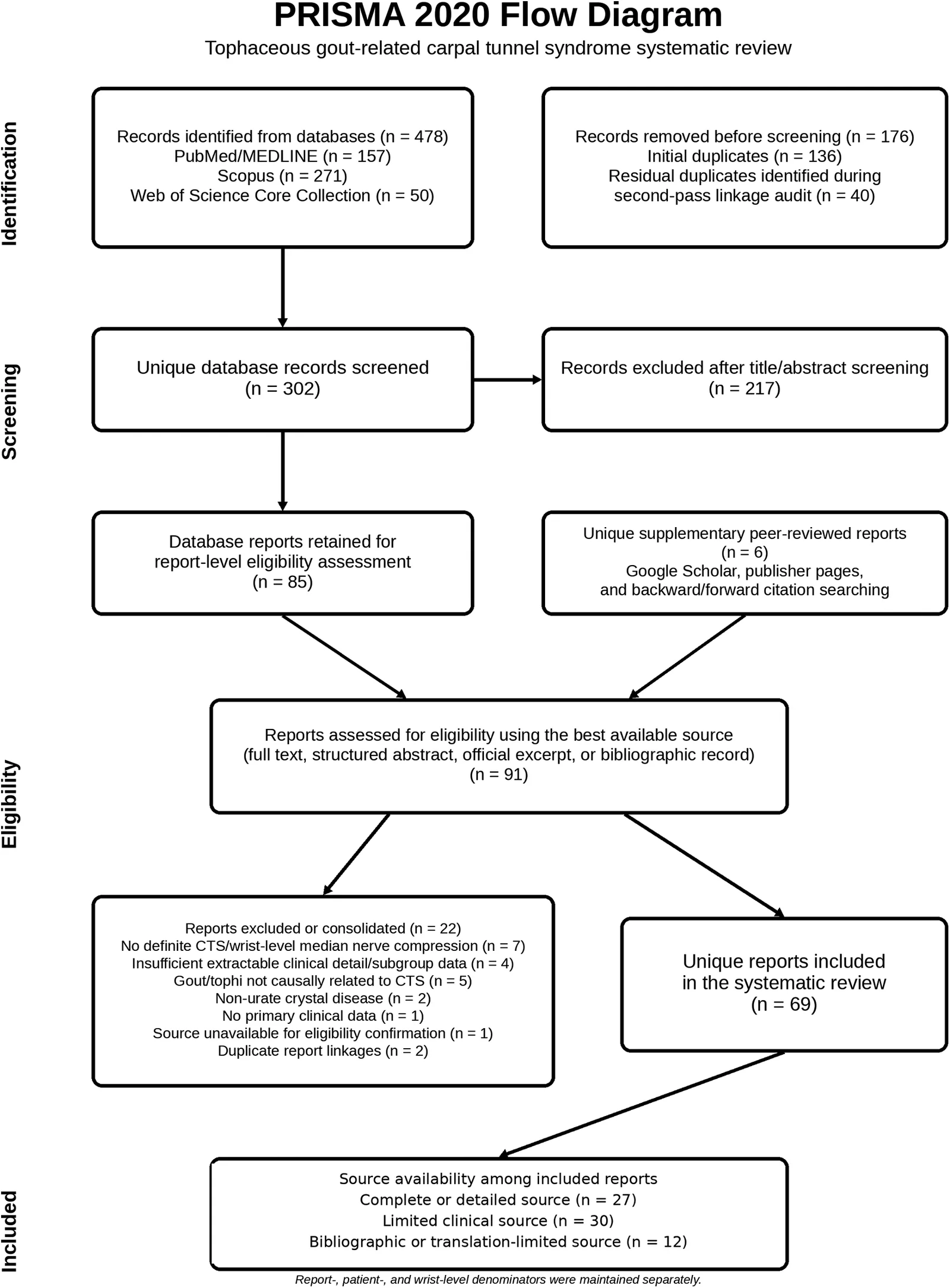
PRISMA 2020 flow diagram. Study identification, screening, eligibility assessment, report consolidation, and source availability for the 69 included canonical reports are shown. The 27 complete/detailed and 30 limited clinical reports formed the primary clinical synthesis and design-appropriate appraisal set (*n* = 57); the 12 bibliographic or translation-limited reports formed a separate historical ancillary set and were not formally JBI-appraised.

The 22 exclusions or consolidations comprised no definite CTS or wrist-level median nerve compression (*n* = 7), insufficient extractable clinical detail or subgroup data (*n* = 4), gout or tophi not causally related to CTS (*n* = 5), non-urate crystal disease (*n* = 2), no primary clinical data (*n* = 1), source unavailable for eligibility confirmation (*n* = 1), and duplicate report linkage (*n* = 2).

### 3.2 Evidence profile and source availability

The canonical evidence map contained 55 case reports or clinical-image reports, 13 case series, and one nonrandomized comparative study. It represented at least 133 patients, 165 CTS wrists, and 159 wrists with explicit gout attribution. These are conservative minimum counts because two historical series did not provide complete CTS-subgroup denominators. Twenty-seven reports had complete or detailed sources, 30 had limited clinical sources, and 12 were bibliographic or translation-limited (Table 1; Supplementary Table S3).

The primary clinical synthesis and design-appropriate appraisal set comprised 57 reports: 44 case reports or clinical-image reports, 12 case series, and one nonrandomized comparative study, representing at least 120 patients, 152 CTS wrists, and 146 explicitly gout-attributable wrists. Causal certainty was Tier 1 in 41 reports, Tier 2 in 5, and Tier 3 in 11. Restricting the synthesis to the 46 Tier 1 + 2 reports and then to the 41 Tier 1 reports did not change the principal qualitative interpretations: flexor tendon or sheath disease, carpal structural disease, and rare direct neural involvement remained represented; open decompression with treatment of the local lesion remained the most frequently reported operative pattern; and comparative evidence remained insufficient to determine superiority of a tendon procedure. These were qualitative robustness checks rather than effect estimates. The remaining 12 reports were retained as historical ancillary evidence only and contributed no unverified clinical outcome claims.

A conservative quantitative descriptive synthesis of the 57-report primary set is presented in Table 1, Panel B. Sex was verifiable for 117 of the minimum 120 patients; 111/117 (94.9%) were male and 6/117 (5.1%) were female. Numeric age information was available in 45/57 reports, with reported ages spanning 20-88 years. Gout or hyperuricemia duration or onset timing was available in 18/57 reports, whereas 34/57 provided a serum-urate value or status and/or patient-specific information on urate-lowering treatment or adherence. Bilateral CTS was explicitly described in 20/57 reports, and visible or peripheral tophi were explicitly documented in 9/57 using a strict clinical definition that excluded imaging-only deposits and nonspecific wrist masses.

Electrodiagnostic testing was explicitly documented in 19/57 reports. At least one named imaging modality was used in 31/57 reports: MRI in 15, radiography in 14, ultrasonography in 7, and CT in 5, including two reports using dual-energy or dual-source CT. Imaging categories were non-mutually exclusive. Local histological, microscopic/crystal, or pathological confirmation of gout was explicitly documented in 19/57 reports. Carpal-tunnel release or decompression was explicitly performed in 34/57 reports; an open, extended-open, or mini-incision approach was specifically described in 18/34 of these reports, whereas the decompression approach was insufficiently specified in the remaining 16/34. A tendon- or tendon-sheath-directed procedure was explicitly reported in 22/57 reports; within this subset, tenosynovectomy or synovectomy was described in 12, debulking, trimming, or debridement in 10, tendon excision or resection in 6, and reconstruction, repair, or tubularization in 7. Tendon-procedure categories were non-mutually exclusive.

### 3.3 Clinical presentation and diagnostic clues

Chronic presentations included nocturnal paresthesia, sensory loss, weakness, thenar atrophy, volar wrist or distal forearm fullness, visible peripheral tophi, restricted finger motion, digital flexion contracture, wrist-level triggering, and recurrent symptoms after previous release (11, 19, 28, 29, 34, 36). Acute or rapidly progressive median neuropathy occurred during gout flares and could present sequentially in both wrists and were managed with urgent decompression in the reported cases (40, 55, 65). Several reports emphasized that the absence of a palpable mass did not exclude covert intratunnel or intratendinous disease (48, 50).

Features most consistently suggesting secondary rather than idiopathic CTS were an unusually severe or rapid course, bilateral or recurrent disease, a history of poorly controlled gout, visible tophi, volar fullness, restricted tendon excursion, and symptoms provoked by finger movement.

### 3.4 Anatomical distribution and mechanisms

Tophaceous disease was reported in the flexor tendon sheaths, flexor tendons, carpal tunnel floor, radiocarpal or other carpal structures, transverse carpal ligament region, lumbricals, and distal forearm. The largest imaging series included 20 male patients and 24 wrists and demonstrated tophaceous deposits in the carpal-tunnel floor (18 wrists), carpal bones (12), radiocarpal joint (12), and extensor tendons or tendon sheaths (11, 26). The latter finding was extensor rather than flexor and was therefore not used as evidence of flexor tendon-sheath deposition.

Flexor tendon involvement ranged from superficial deposits with preserved continuity to diffuse tenosynovitis, fusiform tendon enlargement, extensive intratendinous infiltration, adhesion, erosion, and structural destruction. Flexor digitorum superficialis involvement was most common in recent surgical series, although flexor digitorum profundus and flexor pollicis longus disease also occurred (34, 42, 58, 67). Rare reports described direct median nerve or subepineurial deposition (56). Mechanisms of compression included static mass effect, increased tunnel contents, tendon enlargement constrained beneath the transverse carpal ligament, impaired tendon excursion, inflammatory expansion during a flare, and recurrent compression from residual diseased tendon tissue.

### 3.5 Electrodiagnosis and imaging

Electrodiagnostic testing confirmed median neuropathy and graded its severity in many contemporary reports, although urgent surgery for acute progressive compression was sometimes based on the clinical course and operative findings. MRI was most useful for mapping soft-tissue extent and the relationships among tophi, tendons, and the median nerve. Conventional CT characterized dense deposits and associated bone involvement, whereas dual-source or dual-energy CT supported urate identification in selected cases.

Ultrasonography demonstrated tenosynovial and intratendinous disease, median nerve displacement, and tendon motion. A recent report directly visualized a thickened tophaceous tendon sliding beneath the transverse carpal ligament and reproducing symptoms, illustrating the value of dynamic assessment when symptoms are movement-dependent (68). Imaging findings were not always pathognomonic; intratendinous masses occasionally mimicked neoplasms, supporting multimodal correlation and histopathological confirmation when tissue was obtained.

### 3.6 Management

Open carpal tunnel decompression was the most frequently reported operative approach and permitted direct inspection of the median nerve, flexor sheaths, and tendons. Additional reported procedures included excision or careful debulking of tophi, flexor tenosynovectomy, adhesiolysis, neurolysis, tendon-preserving trimming, selective tendon excision, and reconstruction. Recurrent disease after residual involved tendon tissue was described in individual reports (48). The frequency of open exploration in this literature does not establish superiority over endoscopic or less extensive approaches, which were not directly compared.

Comparative clinical evidence remained limited. Reference 63 included 10 male patients and 13 wrists: 9 wrists underwent tendon-preserving trimming of the involved superficial flexor tendon and 4 underwent tendon resection. Postoperative improvement was reported in both groups; however, the groups were small and not fully matched, the analysis did not account for within-patient clustering, and one wrist treated initially with trimming developed recurrent gouty CTS approximately 1 year later and subsequently underwent resection of the involved superficial flexor tendon (58). These data cannot establish equivalence, superiority, or preference between the procedures.

Reference 72 reported 12 male patients and 14 wrists; all underwent open carpal-tunnel release. Additional wrist-level procedures included tenosynovectomy (72), tendon debulking (4), tendon excision without reconstruction (75), and excision with reconstruction (71), with more than one tendon procedure possible in the same wrist. The Michigan Hand Outcomes Questionnaire score improved from 41.5 to 77.5 at a mean follow-up of 32.6 months. The authors proposed carpal-tunnel release with concomitant tendon debulking as first-line management, excision when the tendon is structurally unsalvageable, and reconstruction when FDP or FPL excision is required (67). A five-patient, seven-wrist series reported improvement after open release and tenosynovectomy or debulking, with no recurrence during short-term follow-up (70). Together, these reports describe anatomy-directed practice and a proposed surgical framework, but they do not establish a universally preferred tendon strategy.

Nonoperative treatment was described in selected stable presentations, including improvement after intensified urate-lowering therapy when surgery was deferred (43, 69). In published reports, acute CTS, progressive neurological loss, a large space-occupying lesion, recurrent compression, or structural tendon destruction were commonly associated with operative exploration; this descriptive pattern should not be interpreted as a validated indication or timing threshold. Sustained urate control and rheumatology follow-up were repeatedly reported across management strategies, although their independent effect on neurological or tendon outcomes cannot be estimated from this evidence. Hypothesis-generating diagnostic and management evidence patterns are summarized in Table 2.

**Table 2:** Hypothesis-generating diagnostic and management evidence patterns.

Clinical context	Reported assessment focus	Evidence pattern/reported management	Evidence caveat
Published demographic pattern	Sex where explicitly reported	111/117 patients with reportable sex were male (94.9%)	Case-based reporting pattern; not a population prevalence estimate or diagnostic criterion
Atypical, severe, bilateral, recurrent, or mass-associated CTS	History of gout, visible tophi, volar fullness, tendon excursion, electrodiagnostic severity	Secondary-cause assessment is relevant when these features are present	Based predominantly on case reports and small series
Movement-dependent symptoms or triggering	Dynamic ultrasound; MRI for anatomical mapping where reported	Dynamic imaging can identify movement-dependent tendon compression in selected cases	Dynamic evidence is limited to isolated reports
Acute or rapidly progressive neurological deficit	Clinical severity and progression; imaging when feasible	Reported acute/progressive cases commonly underwent open decompression and treatment of the local lesion	No comparative timing studies; this is not a validated indication threshold
Diffuse tenosynovitis or discrete tophus	Anatomical mapping of nerve, synovium, and tendon involvement	Release, excision/debulking, and tenosynovectomy were variably reported according to anatomy	Extent of debridement is not standardized and comparative superiority is untested
Intratendinous infiltration	Tendon continuity, residual mechanical strength, and excursion	One 14-wrist retrospective series proposed release with concomitant tendon debulking initially, excision if the involved tendon is unsalvageable, and reconstruction when FDP or FPL excision is required; other reports used preservation/trimming, excision, or reconstruction according to structural findings	Single retrospective series; the proposed algorithm has not been externally validated, and comparative evidence does not establish equivalence, superiority, or a universally preferred tendon procedure
Post-treatment care	Sensory, motor, tendon, and functional follow-up; serum urate and adherence	Hand therapy and urate-lowering management were repeatedly reported components of care	Independent treatment effects and long-term recurrence remain uncertain

### 3.7 Outcomes, recurrence, and adverse events

Most reports with available follow-up described improvement in numbness, pain, motor function, or tendon excursion after the reported intervention. Objective outcome reporting was heterogeneous; only a minority used validated patient-reported measures, grip strength, or repeat electrodiagnostic testing. Follow-up duration varied widely, and recurrence, adherence to hand therapy, and contralateral disease were incompletely documented.

Quantitative follow-up duration was available in 25/57 reports and ranged from 1 to 82 months. Recurrence was conservatively evaluable in 15 reports that provided both a quantitative follow-up interval and an explicit recurrence outcome; postoperative CTS or tophaceous recurrence was described in 2/15. Adverse-event or complication status was explicitly assessable in 25/57 reports, of which 7 described a post-treatment event or complication. One additional report described an intercurrent stroke that was not attributed to surgery. Because these outcomes were reported at heterogeneous patient, wrist, and report levels, these frequencies should not be interpreted as pooled recurrence or complication rates.

The evidence did not establish superiority or equivalence of tendon preservation, trimming, excision, or reconstruction. Preservation or trimming was reported when tendon continuity was retained, whereas excision and reconstruction were reported in cases with destructive infiltration, recurrent disease, or inadequate mechanical integrity. Because treatment selection was strongly confounded by lesion severity and comparative evidence was limited to a small nonrandomized study, the available data are insufficient to determine whether one tendon strategy produces better outcomes than another. Adverse-event reporting was too inconsistent to permit reliable estimation of complication rates.

### 3.8 Critical appraisal

Design-appropriate appraisal was completed for 57 reports. Across the separate design-specific matrices, 481 final item-level judgments were recorded: 324 Yes, 141 Unclear, 6 No, and 10 Not applicable (Supplementary Table S2). This total is presented only as an audit count and is not a pooled cross-design quality measure. The 12 bibliographic or translation-limited historical reports were not formally appraised.

Among 44 case reports or clinical-image reports, takeaway lessons were rated Yes in 43 and diagnostic assessment was adequately described in 38; history and timeline were Unclear in 17, and adverse or unanticipated events were Unclear in 23. Among 12 case series, explicit inclusion criteria were rated Yes in 2, No in 2, and Unclear in 8; consecutive and complete inclusion were each Unclear in 7; outcome or follow-up reporting was Yes in 5, No in 1, and Unclear in 6. The nonrandomized comparative study received 6 Yes, 2 No, and 1 Unclear judgment; the principal concerns were baseline group comparability, incomplete reporting of follow-up completeness, and statistical analysis that did not address clustered bilateral observations. Source-access stratification further showed 17 Unclear judgments among 223 items in the 27 complete/detailed reports (7.6%) vs. 124 among 258 items in the 30 limited clinical reports (48.1%). This contrast supports interpreting many Unclear judgments as limitations of accessible reporting rather than confirmed methodological defects.

## 4 Discussion

### 4.1 Principal findings

This review provides a broader historical and contemporary map of gout-related CTS than previous syntheses. The condition is not a single lesion but a spectrum of synovial, tendinous, osseous, ligament-level, and neural disease. This anatomical heterogeneity explains why presentations range from conventional chronic CTS to acute compression, movement-dependent symptoms, digital contracture, recurrent neuropathy, and multitendon dysfunction.

A further descriptive signal was the marked male predominance of the published evidence. Among the 117 patients for whom sex could be verified, 111 (94.9%) were male. This finding should be interpreted as a characteristic of the available case-based literature rather than as a population prevalence estimate or a validated diagnostic discriminator, because referral, case-selection, and publication patterns may influence the observed distribution.

The evidence map suggests that reported management varied according to the identified compressive tissue and tendon condition. Isolated division of the transverse carpal ligament may not address a documented space-occupying tophus, diffuse tenosynovitis, or markedly infiltrated tendon; however, no comparative evidence establishes the optimal extent of exploration, debridement, or tendon treatment. Reported tendon strategies ranged from preservation or trimming to excision and reconstruction according to structural findings. These recurring patterns are hypothesis-generating and should not be interpreted as prescriptive recommendations or evidence of procedure equivalence or superiority.

### 4.2 Diagnostic implications

A history of gout is an important diagnostic clue but is neither necessary nor sufficient. Some patients presented with CTS as the first recognized manifestation of gout, whereas others had longstanding, poorly controlled disease. Clinically relevant examination features included volar fullness, peripheral tophi, restricted digital motion, triggering at the wrist, and bilateral or recurrent symptoms. Electrodiagnostic testing establishes neuropathic severity but does not identify the causative tissue.

Imaging selection in the reviewed reports varied with the suspected mechanism. Ultrasound was used for superficial and dynamic tendon assessment, MRI for mapping soft-tissue extent, and CT or dual-energy techniques for deposits, bone erosion, or urate characterization. No imaging modality has comparative evidence establishing a universally preferred strategy, and imaging does not replace direct operative or histopathological confirmation when tissue is obtained from a destructive or atypical mass.

### 4.3 Surgical and multidisciplinary implications

Open exploration was the most consistently reported operative strategy and permitted neural decompression together with inspection and treatment of tendon or synovial pathology. Endoscopic release alone would not permit direct treatment of a space-occupying tophus or assessment of tendon destruction, but the literature does not directly compare open, endoscopic, or less extensive approaches; therefore, reporting frequency should not be interpreted as proof of superiority. Acute progressive deficits were treated as time-sensitive compression in the reported cases, but this review cannot define a comparative timing threshold.

A structured anatomy-directed surgical algorithm has been proposed for intratendinous gouty CTS: carpal-tunnel release with concomitant tendon debulking as the initial strategy, tendon excision when the involved tendon is structurally unsalvageable, and reconstruction when FDP or FPL excision is required (67). This framework is clinically informative and directly addresses the tendon pathology emphasized in this review; however, it derives from a small retrospective series and has not been externally validated. Accordingly, the existence of this published algorithm does not establish a universally preferred procedure.

Postoperative rehabilitation and metabolic treatment were repeatedly reported as components of care, but their independent effects cannot be estimated from the available case-based evidence. Extensive tenosynovectomy and tendon procedures were associated conceptually with concerns regarding adhesion and stiffness, while persistent hyperuricemia provides a biological basis for continued deposition; comparative recurrence data are insufficient. Multidisciplinary coordination among hand or reconstructive surgeons, rheumatologists, rehabilitation specialists, and radiologists may be reasonable in severe or recurrent disease, but the review does not test a specific multidisciplinary pathway.

### 4.4 Evidence quality and research priorities

The emergence of recent surgical series improves the evidence base but does not overcome its central limitations. Most data remain case-based, treatment selection is confounded by lesion severity, standardized outcome measures are uncommon, and statistical analyses may ignore bilateral clustering. Future multicenter studies should report patient- and wrist-level denominators separately, define anatomical tendon involvement, document serum urate control and rehabilitation, use validated hand-function measures, report adverse events and recurrence, and account for correlated bilateral observations.

A prospective registry of secondary CTS caused by crystal deposition would be more feasible than a randomized trial and could clarify indications for tendon preservation, excision, and reconstruction. The evidence map in Figure 1 should be viewed as a hypothesis-generating summary of recurring observations rather than a validated algorithm, clinical pathway, or set of treatment indications.

### 4.5 Strengths and limitations of the review

Strengths include searches of three major bibliographic databases with supplementary citation methods, second-pass deduplication and report linkage, independent screening, explicit separation of reports, patients, and wrists, conservative minimum denominators, source-availability stratification, separation of historical ancillary evidence from the primary synthesis, and design-appropriate JBI appraisal without aggregate quality scoring.

The principal limitation is incomplete access to historical and brief reports. Twelve bibliographic or translation-limited reports were retained because the available official source explicitly established causal gout-related CTS; however, they contributed only verified historical facts or conservative minimum counts and were not formally JBI-appraised. This approach improves historical completeness while limiting unsupported inference, but it cannot substitute for complete-text review. Complete texts for some reports may contain additional demographic, treatment, or outcome data.

Other limitations include probable publication bias toward unusual or surgically treated presentations; heterogeneous terminology and diagnostic methods; uncertain subgroup denominators in two historical series; incomplete adverse-event and recurrence reporting; and the absence of meta-analysis or formal certainty grading. Embase was not searched because it was not available through the authors’ institutional subscriptions, and non-English regional databases were not systematically searched. Records indexed uniquely in these sources may therefore have been missed despite searches of PubMed/MEDLINE, Scopus, and Web of Science Core Collection and supplementary structured Google Scholar searching, publisher-page verification, and backward and forward citation searching. The database queries were phrase-based and may also have missed historical reports indexed only under terms such as median neuritis, median neuropathy, or median nerve entrapment; supplementary citation searching mitigated but could not eliminate this risk.

Potential cohort overlap was considered separately from duplicate publication. The reviewer-cited Kaohsiung reports were rechecked and were found to originate from different institutions and author groups (References 31 and 39). However, recruitment periods and institutional detail were incomplete in several historical or source-limited reports, so residual cohort overlap cannot be excluded with certainty; aggregate patient and wrist totals should therefore be interpreted as conservative minimum estimates.

Several eligible reports were published in outlets not indexed in MEDLINE. Indexing status was not used as a proxy for methodological quality; instead, source accessibility and design-appropriate JBI appraisal were evaluated separately. Nevertheless, variability in indexing, source accessibility, and reporting standards contributes to uncertainty in portions of the evidence base. The review was not prospectively registered. The full database strategies, final report-level exclusion and linkage decisions, canonical extraction dataset, quantitative descriptive-synthesis output, and item-level critical appraisal matrices are supplied to support reproducibility; however, database indexing and source-access status may change over time.

## 5 Conclusion

Tophaceous gout is a rare but important cause of secondary CTS, with disease ranging from diffuse tenosynovitis and preserved tendon continuity to extensive intratendinous destruction and direct median nerve involvement. Atypical, acute, severe, recurrent, bilateral, or mass-associated CTS may warrant targeted assessment for gout and space-occupying pathology. In surgically treated reports, decompression was commonly combined with anatomy-directed management of tophaceous and tendon disease, followed by rehabilitation and urate control. These observations are hypothesis-generating: current evidence is insufficient to define validated treatment indications, a preferred decompression approach, or superiority or equivalence among tendon-preserving, excisional, and reconstructive procedures.

## Data Availability

The original contributions presented in the study are included in the article/Supplementary Material, further inquiries can be directed to the corresponding author.
