# Supplementary material for: Tophaceous gout-related carpal tunnel syndrome and flexor tendon involvement: a systematic review
Source: Front Surg. 2026 Oct 2;13:1948883. doi: 10.3389/fsurg.2026.1948883 (PMC13714934; doi:10.3389/fsurg.2026.1948883)
Supplement: Supplementary file 1 [file Table1.docx]

**Supplementary Table S1. Search Strategies, PRISMA Accounting, and Report-Level Exclusions**

Final search update: 23 July 2026. No date or language filters were applied in the final searches. The prespecified search plan included PubMed/MEDLINE, Scopus, and Web of Science Core Collection but not Embase. Embase was not available through the authors' institutional subscriptions during the review period and therefore could not be searched reproducibly. Non-English regional databases were not systematically searched. Dynamic Google Scholar total-hit estimates were not used in PRISMA accounting; only unique peer-reviewed reports entered into the screening log were counted. ACG and MSO independently completed title/abstract screening and report-level eligibility assessment. One reviewer entered extraction data and the second independently verified every populated and NR field against the best available source; discrepancies were resolved by consensus.

# A. Database and supplementary search strategies

| **Source** | **Coverage / date** | **Final reproducible strategy** | **Records entering database denominator** |
| --- | --- | --- | --- |
| PubMed/MEDLINE | Inception to 23 July 2026 | (("carpal tunnel syndrome"[Title/Abstract] OR "median nerve compression"[Title/Abstract]) AND (gout[Title/Abstract] OR tophaceous[Title/Abstract] OR tophus[Title/Abstract] OR "monosodium urate"[Title/Abstract]) AND (wrist[Title/Abstract] OR hand[Title/Abstract] OR flexor[Title/Abstract] OR tendon[Title/Abstract] OR tenosynovitis[Title/Abstract])) | 157 |
| Scopus | Inception to 23 July 2026 | TITLE-ABS-KEY(("carpal tunnel syndrome" OR "median nerve compression") AND (gout OR tophaceous OR tophus OR "monosodium urate") AND (wrist OR hand OR flexor OR tendon OR tenosynovitis)) | 271 |
| Web of Science Core Collection | Inception to 23 July 2026 | TS=(("carpal tunnel syndrome" OR "median nerve compression") AND (gout OR tophaceous OR tophus OR "monosodium urate") AND (wrist OR hand OR flexor OR tendon OR tenosynovitis)) | 50 |
| Google Scholar / publisher pages / citation searching | Updated through 23 July 2026 | Phrase combinations included: "carpal tunnel syndrome" gout tophus; "median nerve compression" gout wrist; "carpal tunnel" "flexor tendon" gout; "acute carpal tunnel syndrome" gout. Publisher-page verification and backward/forward citation searching were conducted for included reports and relevant reviews. | 6 unique supplementary peer-reviewed reports |

# B. PRISMA accounting

| **Stage** | **Count** | **Accounting note** |
| --- | --- | --- |
| Database records identified | 478 | PubMed 157 + Scopus 271 + Web of Science 50 |
| Duplicates removed | 176 | 136 initial duplicates + 40 residual duplicates identified during second-pass linkage audit |
| Unique database records screened | 302 | Independent ACG and MSO title/abstract screening |
| Database records excluded | 217 | Final consensus after independent screening |
| Database reports retained | 85 | Advanced to report-level assessment |
| Unique supplementary peer-reviewed reports | 6 | Entered report-level assessment after deduplication |
| Reports assessed using best available source | 91 | Complete text, detailed abstract, official excerpt, or bibliographic/indexing record |
| Reports excluded | 20 | See section C |
| Duplicate report representations consolidated | 2 | Alternate-title or truncated-author representations of canonical reports 71 and 75 |
| Unique reports included | 69 | 55 case reports / clinical-image reports, 13 case series, and 1 nonrandomized comparative study |

# C. Reports excluded after report-level assessment or consolidated as duplicate representations

| **Full-report no.** | **Year** | **Report title** | **Final action** | **Primary reason** |
| --- | --- | --- | --- | --- |
| 1 | 2026 | Causal effects of type 2 diabetes, obesity, gout, and hypothyroidism on carpal tunnel syndrome: a univariable and multivariable Mendelian randomization study. | Excluded | No primary clinical data |
| 6 | 2025 | [Clinical observation of free palmaris longus tendon graft reconstruction in treatment of gouty tophus erosion lesions in flexor tendon of wrist and hand]. | Excluded | No definite CTS or wrist-level median nerve compression |
| 7 | 2024 | Surgical Management of Tophaceous Gout in the Upper Limb | Excluded | Insufficient clinical detail for an extractable gout-related CTS subgroup |
| 13 | 2023 | Gouty tenosynovitis with compartment syndrome in the hand: A case report. | Excluded | No definite CTS or wrist-level median nerve compression |
| 17 | 2022 | The Use of Ultrasound to Differentiate Between Gout and Infection in the Wrist | Excluded | No definite CTS or wrist-level median nerve compression |
| 22 | 2019 | Atypical musculoskeletal manifestations of gout in hyperuricemia patients. | Excluded | Gout/tophi not causally related to CTS |
| 26 | 2018 | Analysis of clinical effect of surgical treatment in patients with multiple tophi in extremities: A report of 23 cases | Excluded | No definite CTS or wrist-level median nerve compression |
| 28 | 2017 | Chronic tophaceous gout. | Excluded | No definite CTS or wrist-level median nerve compression |
| 38 | 2012 | Unusual causes of carpal tunnel syndrome: space occupying lesions. | Excluded | Insufficient clinical detail for an extractable gout-related CTS subgroup |
| 43 | 2009 | Crystal deposition disease masquerading as proliferative tenosynovitis and its associated sequelae. | Excluded | Insufficient clinical detail for definite gout-related CTS |
| 44 | 2009 | Carpal tunnel syndrome caused by space occupying lesions. | Excluded | Insufficient clinical detail for an extractable gout-related CTS subgroup |
| 46 | 2007 | Watch out for 'pins and needles' in hands--it may be a case of gout. | Excluded | Full text unavailable for eligibility confirmation |
| 49 | 2003 | Gouty involvement of flexor tendons. | Excluded | No definite CTS or wrist-level median nerve compression |
| 52 | 2001 | Gouty tenosynovitis of the wrist. | Excluded | No definite CTS or wrist-level median nerve compression |
| 56 | 1999 | Case report. Initial manifestation of gout after carpal tunnel release. | Excluded | Gout/tophi not causally related to the preoperative CTS |
| 61 | 1993 | Carpal tunnel release complicated by acute gout. | Excluded | Gout/tophi not causally related to the preoperative CTS |
| 63 | 1992 | Acute gout after carpal tunnel release. | Excluded | Gout/tophi not causally related to the preoperative CTS |
| 70 | 1985 | Acute median nerve compression. | Excluded | Gout/tophi not causally related to CTS |
| 78 | 1976 | An uncommon surgical emergency: carpal tunnel syndrome due to chalky gout | Excluded | Non-urate calcium-crystal disease (Kalkgicht/calcium gout; chondrocalcinosis/CPPD spectrum), outside the monosodium-urate gout eligibility criterion. |
| 79 | 1976 | [A rare surgical emergency: calcium gout complicated by acute carpal tunnel]. | Excluded | Non-urate calcium-crystal disease (Kalkgicht/calcium gout; chondrocalcinosis/CPPD spectrum), outside the monosodium-urate gout eligibility criterion. |
| 68 | 1985 | The carpal tunnel syndrome in gout | Consolidated | Duplicate representation linked to an existing canonical report; not counted separately |
| 73 | 1982 | A case of carpal tunnel syndrome caused by tophaceous gout | Consolidated | Duplicate representation linked to an existing canonical report; not counted separately |

# D. Source-availability rules for included reports

| **Source category** | **Included reports** | **Permitted contribution** | **Safeguard against overinterpretation** |
| --- | --- | --- | --- |
| Complete or detailed source | 27 | Primary clinical synthesis and design-appropriate JBI appraisal; all explicitly supported clinical, anatomical, treatment, and outcome variables | No inference beyond the accessible source |
| Limited clinical source | 30 | Primary clinical synthesis and design-appropriate JBI appraisal; only variables explicitly supported by the accessible abstract, publisher excerpt, or indexed clinical text | Unavailable fields coded NR; source access is recorded separately from appraisal and is not interpreted as methodological weakness |
| Bibliographic or translation-limited source | 12 | Historical ancillary evidence and conservative minimum-count facts only; no formal JBI appraisal | No unverified clinical, treatment, outcome, recurrence, adverse-event, or appraisal claim |

Interpretation note: The 27 complete/detailed and 30 limited clinical reports formed the primary clinical synthesis and design-appropriate JBI appraisal set (n = 57; 44 case reports/clinical-image reports, 12 case series, and 1 nonrandomized comparative study). A limited clinical source was an official abstract, publisher excerpt, or indexed clinical text sufficient to establish eligibility and selected variables when no complete text was reviewed. The 12 bibliographic/translation-limited reports were retained separately as historical ancillary evidence and conservative minimum counts and were not formally JBI-appraised. Source limitation was not treated as evidence that a feature was absent or methodologically deficient; inaccessible fields were coded NR and were not inferred. The primary set represented at least 120 patients, 152 CTS wrists, and 146 explicitly gout-attributable wrists; the full 69-report historical evidence map represented conservative minimums of 133 patients, 165 CTS wrists, and 159 gout-attributable wrists.

# E. Causal-certainty classification and sensitivity-analysis rules

| Causal-certainty category | Reports | Operational definition | Sensitivity role |
| --- | --- | --- | --- |
| Tier 1 — direct local confirmation | 41 | Definite CTS/wrist-level median neuropathy plus direct operative visualization/removal and/or histopathological or crystal confirmation of the local compressive gouty lesion. | Included in primary synthesis; direct-confirmation sensitivity set. |
| Tier 2 — anatomically supported | 5 | Objective CTS/median neuropathy plus imaging-localized gout/tophi anatomically linked to median-nerve compression without direct local pathological/crystal confirmation in the accessible source. | Included in primary synthesis and Tier 1+2 stronger-confirmation sensitivity set. |
| Tier 3 — explicit report-level attribution | 11 | Primary report explicitly attributes CTS/wrist-level median neuropathy to gout/tophi, but accessible source does not expose direct local or sufficiently specific anatomical imaging confirmation. | Included in primary synthesis; excluded from stronger-confirmation sensitivity sets. |
| Historical ancillary — not graded | 12 | Bibliographic or translation-limited evidence retained only for verified historical facts and conservative minimum counts. | Not included in causal-certainty sensitivity analysis. |

Sensitivity-analysis note: A post hoc narrative sensitivity analysis was repeated qualitatively after excluding Tier 3 reports (Tier 1+2, n = 46) and after restricting to Tier 1 reports (n = 41). These robustness checks did not generate pooled effect estimates or imply equivalence or superiority of treatment strategies.

**F. Additional reproducibility safeguards**

The following safeguards were applied or clarified during peer review. They did not change report eligibility or the locked extracted clinical data.

| **Check** | **Method / rule** | **Result / safeguard** |
| --- | --- | --- |
| **Quantitative descriptive synthesis** | Variable-specific patient- or report-level denominators were calculated from the 57-report primary clinical synthesis. NR fields were excluded and not imputed; imaging and tendon-procedure subcategories were allowed to overlap. | The resulting conservative reporting frequencies are presented in main Table 1, Panel B and Supplementary Table S3C. They are not interpreted as prevalence, treatment-effect, recurrence-incidence, or complication-incidence estimates. |
| **Potential cohort overlap** | Potential overlap beyond duplicate publication was assessed, where source detail permitted, using institution, authorship, recruitment period, patient/wrist counts, demographic characteristics, and intervention details. | The Kaohsiung reports cited as References 31 and 39 originated from different institutions and author groups. No definite additional overlap was identified where source detail was adequate; residual overlap cannot be excluded in some historical/source-limited reports, so aggregate patient and wrist counts remain conservative minimum estimates. |
| **Indexing and peer-review eligibility** | MEDLINE indexing status, journal impact metrics, and journal prestige were not used as eligibility criteria. Preprints and conference abstracts without sufficient peer-reviewed clinical information were excluded. | Indexing status was not used as a proxy for methodological quality. Source accessibility and design-appropriate JBI appraisal were evaluated separately. |
| **Search-coverage limitation** | Embase was not available through the authors' institutional subscriptions and was not searched. Non-English regional databases were not systematically searched. | Records indexed uniquely in those sources may have been missed despite PubMed/MEDLINE, Scopus, Web of Science Core Collection, structured Google Scholar searching, publisher-page verification, and backward/forward citation searching. |
